# Regulatory properties of vitronectin and its glycosylation in collagen fibril formation and collagen-degrading enzyme cathepsin K activity

**DOI:** 10.1038/s41598-021-91353-6

**Published:** 2021-06-08

**Authors:** Kimie Date, Hiromi Sakagami, Kei Yura

**Affiliations:** 1 Institute for Human Life Innovation, Ochanomizu University, 2-1-1 Otsuka, Bunkyo-ku, Tokyo, 112-8610 Japan; 2 Faculty of Science, Department of Chemistry, Ochanomizu University, 2-1-1 Otsuka, Bunkyo-ku, Tokyo, 112-8610 Japan; 3 Graduate School of Humanities and Sciences, Ochanomizu University, 2-1-1 Otsuka, Bunkyo-ku, Tokyo, 112-8610 Japan; 4 Center for Interdisciplinary AI and Data Science, Ochanomizu University, 2-1-1 Otsuka, Bunkyo-ku, Tokyo, 112-8610 Japan; 5 School of Advanced Science and Engineering, Waseda University, 513 Tsurumaki, Waseda, Shinjuku-ku, Tokyo, 162-0041 Japan

**Keywords:** Biochemistry, Molecular biology

## Abstract

Vitronectin (VN) is a glycoprotein found in extracellular matrix and blood. Collagen, a major extracellular matrix component in mammals, is degraded by cathepsin K (CatK), which is essential for bone resorption under acidic conditions. The relationship between VN and cathepsins has been unclear. We discovered that VN promoted collagen fibril formation and inhibited CatK activity, and observed its activation in vitro. VN accelerated collagen fibril formation at neutral pH. Collagen fibers formed with VN were in close contact with each other and appeared as scattered flat masses in scanning electron microscopy images. VN formed collagen fibers with high acid solubility and significantly inhibited CatK; the IC_50_ was 8.1–16.6 nM and competitive, almost the same as those of human and porcine VNs. VN inhibited the autoprocessing of inactive pro-CatK from active CatK. DeN-glycosylation of VN attenuated the inhibitory effects of CatK and its autoprocessing by VN, but had little effect on acid solubilization of collagen and VN degradation via CatK. CatK inhibition is an attractive treatment approach for osteoporosis and osteoarthritis. These findings suggest that glycosylated VN is a potential biological candidate for CatK inhibition and may help to understand the molecular mechanisms of tissue re-modeling.

## Introduction

Vitronectin (VN), a glycoprotein present in extracellular matrix (ECM) and blood, is found in various organs including the bone matrix and liver, the latter of which has its highest abundance^1–3^. The normal VN concentration in human plasma is approximately 200–400 μg/mL, and it is reduced in patients with liver disease^4,5^. VN plays important roles in cell adhesion, spreading, migration, and wound healing, since it binds with major ECM receptors, integrins, and other ECM components such as collagen and proteoglycans. VN also regulates blood coagulation, complement activity, and the fibrinolytic system^6^. Six purified animal plasma VNs contain 10–20% carbohydrates by mass, and N-linked oligosaccharides are present in all six species of VNs as detected using a lectin binding assay^7^. We have previously shown that the glycosylation of VNs changes during liver regeneration and cirrhosis^5,8,9^. In addition, it has been demonstrated that deN-glycosylation and desialylation of VNs in humans, swine, and rodents increase collagen-binding activity^5,8,10^. Desialylated VN (deNeu-VN), but not deN-glycosylated VN (deN-gly-VN), remarkably decreases the spread of dermal fibroblasts and hepatic stellate cells compared to control VN^10,11^. These findings suggest that VN and its glycosylation should play crucial roles in re-modeling of organs and tissues containing collagen as the main component. Previous studies have focused on the cell adhesion–promoting activity of VN in osteoporosis^12–14^. The effects of VN on collagen fibril formation and acid solubilization are, however, still little understood. Additionally, there are no published reports regarding the effects of VN on physiological and pathological organic bone matrix degradation via the action of osteoclast proteases such as cathepsin K (CatK, RefSeq NM_000396.4). Moreover, it is unclear whether VN glycosylation affects fibril formation and collagen degradation during tissue re-modeling.

Collagen is a major ECM component in mammals. Tissue is metabolized and maintained according to the balance between collagen degradation and formation. Type I collagen represents approximately 90% of the total protein in organic bone matrix, and forms fibrils in the bone matrix^15^. During bone formation, collagen molecules spontaneously form fibrils of aligned protein helices with non-collagenous protein such as osteopontin^16–18^, and on which tiny crystals of hydroxyapatite, an essential mineral of human bone composed of inorganic compounds, can grow^19^. Interaction between collagen, non-collagenous protein, and hydroxyapatite is critical to bones’ resilience and strength^17,20^. In bone degradation, osteoclasts secrete acid and acid protease. Bone mineral from collagen fibril^21^ is dissolved in this acidic milieu. Collagen-formed fibril in the organic bone matrix is solubilized^22^ and degraded^23^. Therefore, it is crucial to clarify VN’s effects on collagen fibril formation and degradation by CatK to understand VN’s the role in bone re-modeling.

Bone diseases such as osteoporosis are associated with age-related declines in collagen levels in the bone, and possible treatments to counteract the collagen loss are being studied. The treatment of these bone diseases focuses on promoting collagen formation and/or suppressing collagen degradation. CatK, a cysteine protease active at low pH, is predominantly expressed in osteoclasts and secreted as a proenzyme. It is essential in the degradation of type I collagen in organic bone matrix^24,25^. Pro-CatK, an inactive proenzyme of CatK, is a precursor with 329 amino acid residues, including a 15-amino acid pre-region, a 99-amino acid pro-region, and a mature active enzyme comprising 215 amino acid residues. It is activated to generate mature active CatK by autocatalytic removal of its N-terminal pro-region at low pH^26^. CatK inhibitors have been developed as therapeutic candidates for patients with postmenopausal osteoporosis. CatK inhibitors have been shown to have effect on overcoming the side effects of inhibiting bone formation in clinical trials^27–29^. However, the most anticipated CatK inhibitor, odanacatib, has not been approved owing to another unexpected side effect such as stroke^30^. The underlying cause of the side effect is unknown, because only a few biomolecules have been found to interact with CatK. Another CatK inhibitor, MIV-711, has recently shown efficacy and safety for osteoarthritis treatment in clinical trials and is currently under development^31,32^.

In this study, we identified VN as a potential biomolecule to inhibit CatK activity. VN and its glycosylation had the following effects on in vitro experiments evaluating matrix formation and degradation during bone re-modeling. First, VN accelerated collagen fibril formation and induced morphological changes in collagen fibrils. Second, VN enhanced the acidic solubility of fibril collagen. Third, VN significantly inhibited not only CatK activity, but also its autoprocessing. Fourth, VN glycosylation inhibited CatK activity and its autoprocessing.

## Results

### VN affects collagen fibril formation

Type I collagen accounts for approximately 90% of the whole protein in the organic bone matrix and forms fibrils in bone^15^. First, to clarify the effects of VN on collagen fibril formation, the turbidities of collagen solutions in the presence or absence of VN were measured. Although turbidity does not directly reflect fibril formation, increased turbidity indicates changes in collagen fibril and fibril formation. Therefore, this assay is widely used as a simple and effective method to identify factors influencing fibril formation in solution^33,34^. The turbidity measurement of the collagen solution generates a sigmoid curve composed of a lag phase, a subsequent growth period, and a plateau that indicates complete gelation due to warming^33,34^.

**Figure 1:**
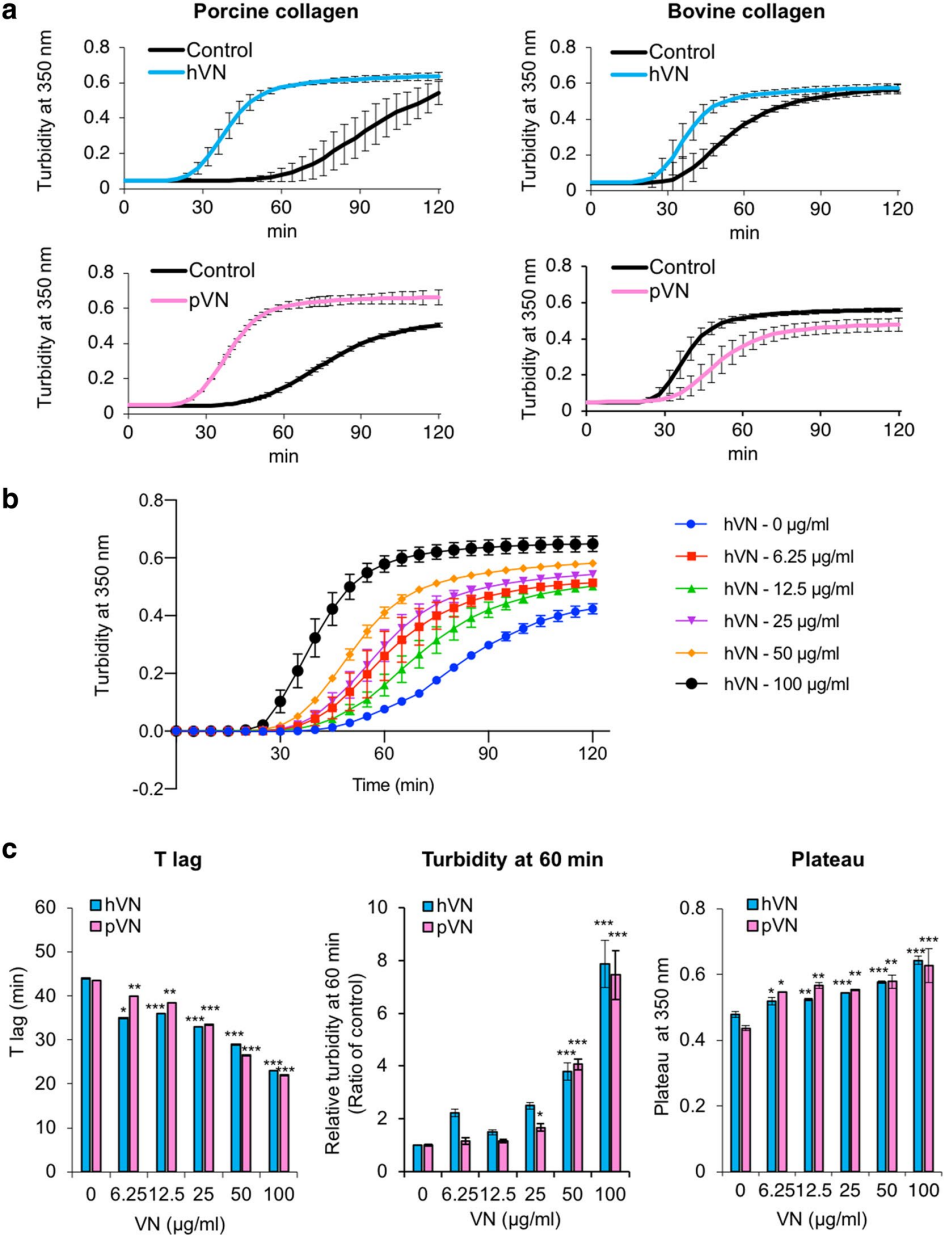
Effects of VNs on collagen fibril formation. (**a**) Time courses of collagen fibril formation in the absence or presence of 0.1 mg/mL hVN or pVN at 37 °C at pH 7.2. Black line is the control; blue line is hVN; pink line is pVN. Left panels show fibril formation of porcine collagen. Right panels show fibril formation of bovine collagen. (**b**) Time courses of porcine collagen fibril formation with indicated concentration of hVN at 37 °C and pH 7.2. (**c**) Dose-dependence of hVN and pVN for T lag, turbidity at 60 min, and plateau of porcine collagen fibril formations. Blue bars are hVN; pink bars are pVN. *p < 0.05; **p < 0.01; ***; < 0.001 compared with 0 μg/mL VN as control by one-way ANOVA with Dunnett's post-hoc test. n = 4. Values represent the mean, and error bars represent the SE. Experiments were performed independently 3–4 times.

**Figure 2:**
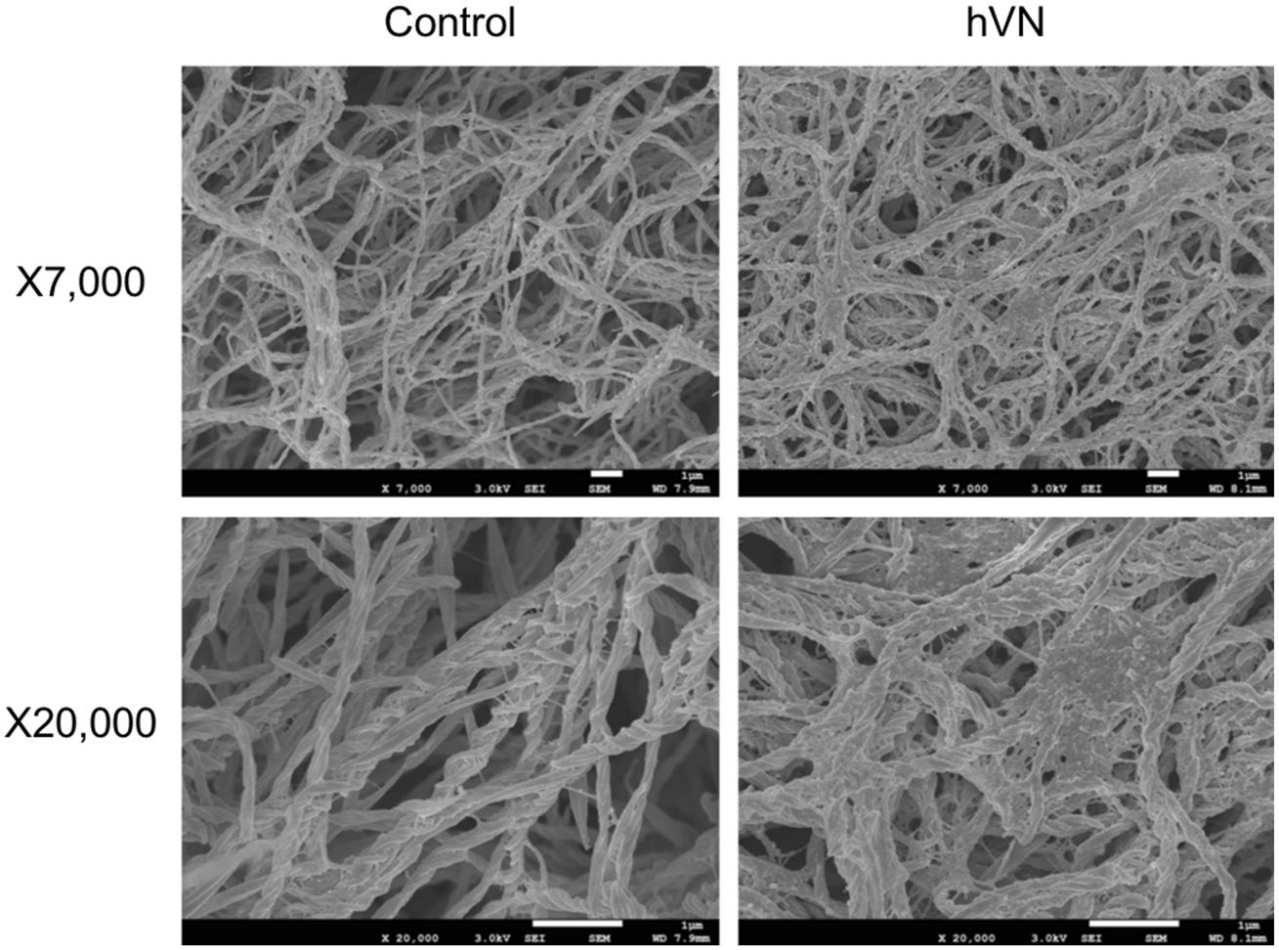
VN-induced morphological changes in collagen fibers. Porcine collagen (1 mg/mL), with or without 0.1 mg/mL hVN in TBS, was incubated at 37 °C for 2 h in a 96 well plate. Collagen fibrils formed in the well were collected and fixed in a tube. The samples were dehydrated, frozen, coated with osmium, and then observed by SEM at ×7000 and ×20,000 magnifications. Scale bar 1 μm. Representative photos of the three.

Human VN (hVN) induced rapid fibril formation in porcine and bovine collagen (Fig. 1a). Moreover, porcine VN (pVN) accelerated fibril formation in porcine collagen but inhibited it in bovine collagen. Interestingly, hVN enhanced porcine collagen fibril formation in a dose-dependent manner (Fig. 1b). Both hVN and pVN prolonged T lag, increased turbidity at 60 min, and increased plateau turbidity in Fig. 1c., suggest that VN increased the collagen fibril formation rate and the molecular weight or thickness of the aggregate^33,34^. Collagen fibrils formed in the absence or presence of hVN was observed via scanning electron microscopy (SEM) at both low magnification (7000×) and high magnification (20,000×). Collagen fibers formed in the presence of VN were in closer contact with each other than those formed in the absence of VN, and clumps were randomly present (Fig. 2). Pore areas of the collagen fibers of control and hVN were 5.62 ± 2.57μm^2^ and 1.02 ± 0.47μm^2^, respectively. These results show that hVN accelerates collagen fibril formation and changes the morphology of collagen fibers, but pVN acts differently depending on the collagen species.

### Collagen fibers formed with VN are easily acid-solubilized

**Figure 3:**
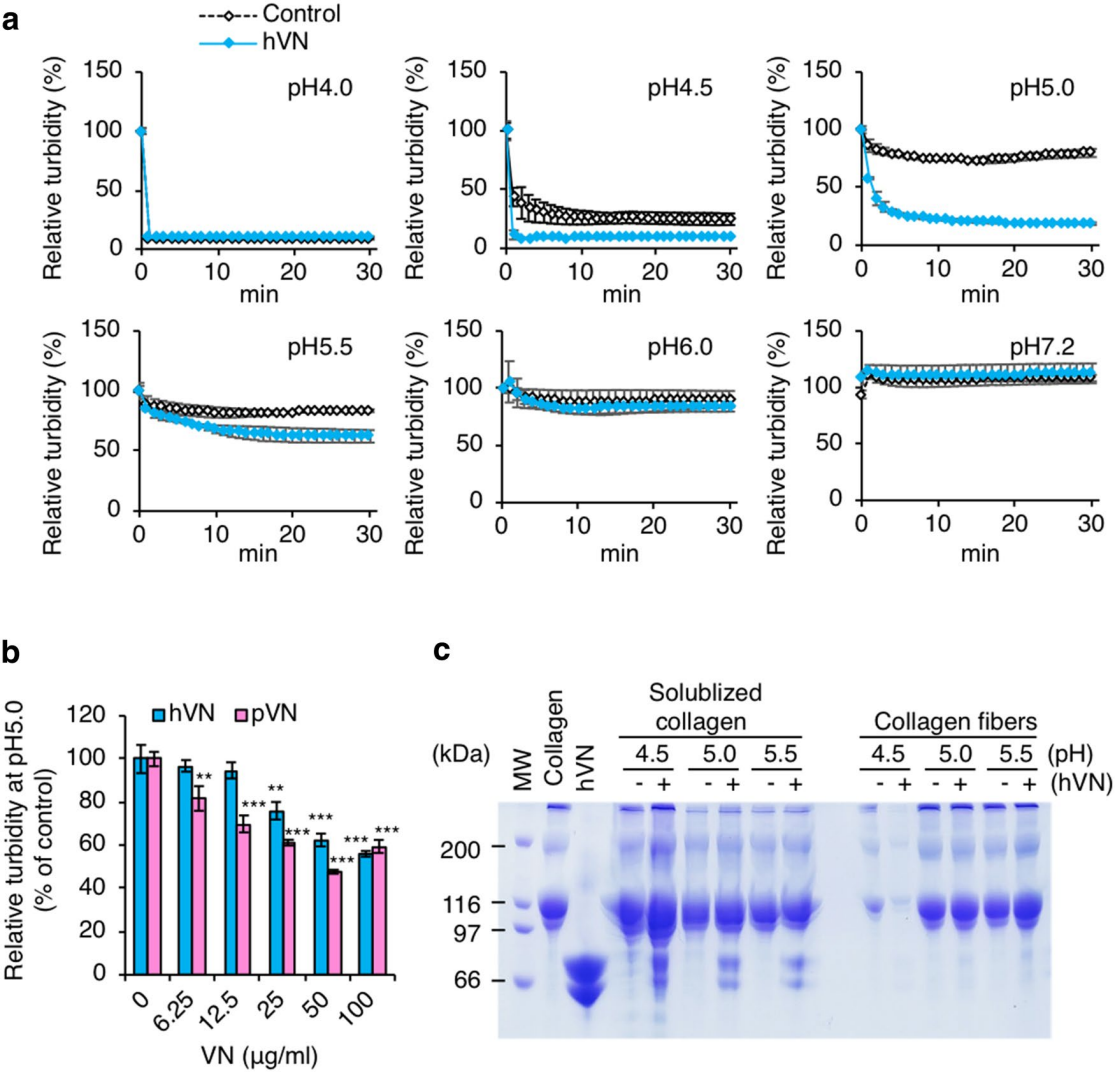
Effects of VNs on acid solubilization of collagen fibers. (**a**) Acid solubilization of porcine collagen fibers formed with 0.1 mg/mL hVN. Solubilizations of collagen fibers were measured at 350 nm and are indicated as relative turbidity (% of 0 min). White diamonds are control; blue diamonds are hVN. (**b**) Dose-dependence of hVN and pVN on relative turbidity of porcine collagen solution at pH 5.0 and 20 min. Blue bars are hVN; pink bars are pVN. **p < 0.01; ***p < 0.001 compared with 0 μg/mL VN as control by one-way ANOVA with Dunnett's post-hoc test. n = 4. (**c**) SDS-PAGE of solubilized collagen and collagen fibers in porcine collagen samples after acid solubilization measurements in the indicated pH for 30 min. MW, molecular weight; untreated collagen 10 μg/lane; hVN 2 μg/lane; -, without 0.1 mg/mL hVN; + , with 0.1 mg/mL hVN. Values represent the mean, and error bars represent the SE. Experiments were performed independently 3–4 times.

Collagen fibers are formed at neural pH and dissolve under acidic conditions. Collagen is degraded by CatK under acidic conditions. Therefore, the collagen in bone is likely solubilized by large amounts of acid secretion from osteoclasts during and/or before degradation by CatK^35^. To determine the effects of VN on the acid solubilization of collagen fibers, the decrease in turbidity was measured using an in vitro assay. Figure 3a shows that the turbidity of porcine collagen fibers formed in neutral pH significantly decreased to 10% and 25% at pH 4.0 and 4.5, respectively, in the control, which did not contain hVN. In contrast, the turbidity did not change at pH ≥ 5.0 compared to the turbidity at 0 min before the pH change. In the presence of hVN, the turbidity of the collagen fibers decreased to approximately 10% at pH 4.0 and 4.5, and to approximately 19% at pH 5.0. Moreover, the pH dependency of pVN and hVN were similar. The turbidity of collagen fibrils formed in the presence of hVN or pVN decreased in a concentration-dependent manner, and pVN reduced turbidity at slightly lower concentrations than hVN did (Fig. 3b). The molecular weight of hVN is 75 kDa, that of nicked product is 65 kDa, and that of pVN is 58 kDa. Thus, the molarities of hVN and pVN at 0.1 mg/mL were 1.3 μM and 1.7 μM, respectively. Notably, hVN increased collagen solubilization and decreased the content of collagen fibers at pH 4.5 (Fig. 3c). Moreover, hVN was mainly found in the solubilized collagen fraction and had the same effect on bovine collagen fibers as on the porcine collagen fibers; pVN also showed effects similar to those of hVN (Supplementary Figure S1). These results showed that the collagen fibers formed in the presence of VN were easily solubilized in acid. This property was common to both hVN and pVN, which exerted similar effects on porcine-derived and bovine-derived collagen fibers. Since both collagen and VN are ECM components and collagen is a well-known ligand for VN, we expected that VN would form acid-resistant collagen via strong interactions between VN and collagen, and hence the current results were unexpected.

### VN inhibits CatK activity

**Figure 4:**
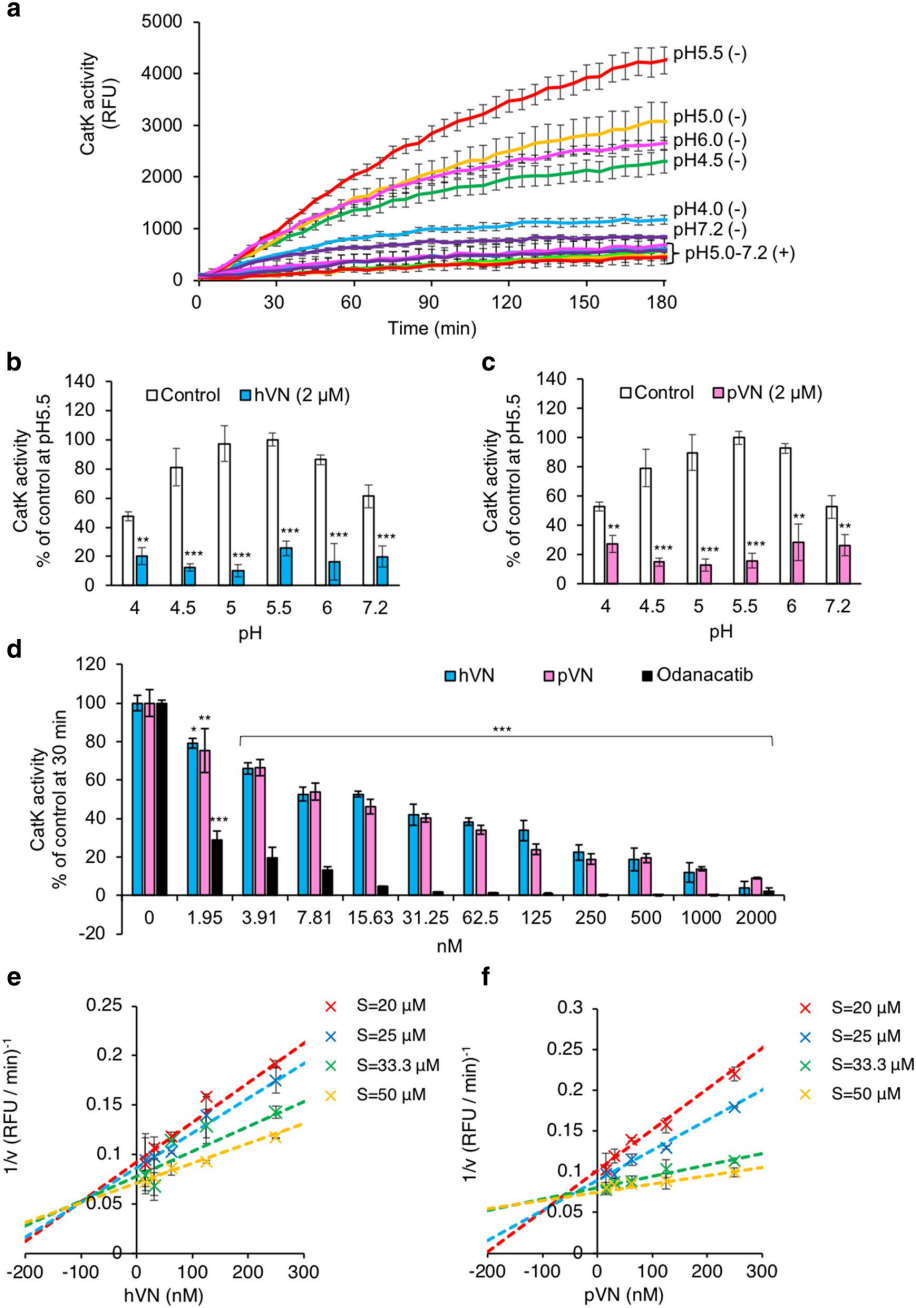
Effects of VNs on CatK activity. CatK activity was measured using a fluorogenic substrate in 96-well plates for 180 min. Fluorescence intensity is measured as relative fluorescence units (RFU). (**a**) Time-dependent effects of hVN (2 μM). (−), without hVN (2 μM); (+), with hVN (2 μM). Values represent the mean, and error bars indicate standard deviation. (**b**,**c**) Effects of 2 μM hVN (**b**) or pVN (**c**) under pH 4.0–7.2 at 30 min. CatK activities are shown as % of the control at pH 5.5. **p < 0.01 and ***p < 0.001 compared with the control via an unpaired *t* test. n = 5. (**d**) Dose-dependence of hVN, pVN, and odanacatib at pH 5.5. CatK activities are shown as % of control at 30 min. *p < 0.05; **p < 0.01; ***p < 0.001 compared with 0 nM hVN, pVN, or odanacatib as a control using one‐way ANOVA with Dunnett's post‐hoc test. n = 4–6. (**e**,**f**) Dixon plot between VNs and CatK. Substrates were 20, 25, 33.3, and 50 μM. hVN (**e**) or pVN (**f**) were 0, 3.91, 7.81, 15.6, 31.3, 62.5, 125, or 250 nM. Values represent the mean, and error bars represent the SE. Experiments were performed independently 3–6 times.

The potential suppressive effects of VNs on the activity of CatK, which degrades type I collagen under acidic conditions, were examined using a fluorescence-labeled substrate. CatK activities at pH 4.0–7.2 were measured and found to be the highest at pH 5.5 in the absence of hVN (−) (Fig. 4a). The optimal pH for CatK is 5.5, which is consistent with our results. Surprisingly, hVN (+) strongly inhibited CatK activity at all pH values. The inhibition of CatK with 2 μM hVN was especially strong at pH 4.5–7.2 (Fig. 4b). Moreover, pVN strongly inhibited CatK activity at pH 4.5–5.5, similar to hVN (Fig. 4c). Both hVN and pVN dose-dependently inhibited CatK activity at pH 5.5. At 2000 nM, hVN and pVN inhibited CatK activity by 6.05% and 9.93%, and their IC_50_ values were 16.57 ± 46.33 nM and 8.13 ± 11.47 nM, respectively (Fig. 4d). The IC_50_ value of odanacatib, a CatK inhibitor, was 1.24 ± 0.46 nM. In the Dixon plot, CatK inhibition by hVN and pVN resulted in a line crossing into the negative values on the X-axis (Fig. 4e,f). These observations provide evidence that VNs can competitively inhibit CatK activity at a concentration approximately 240–640 times lower than that in blood, despite having a weaker inhibitory effect than odanacatib. It was surprising that VNs inhibited CatK activity, as there is no report of the relationship between VNs and cathepsins.

### VN inhibits autoprocessing of pro-CatK

**Figure 5:**
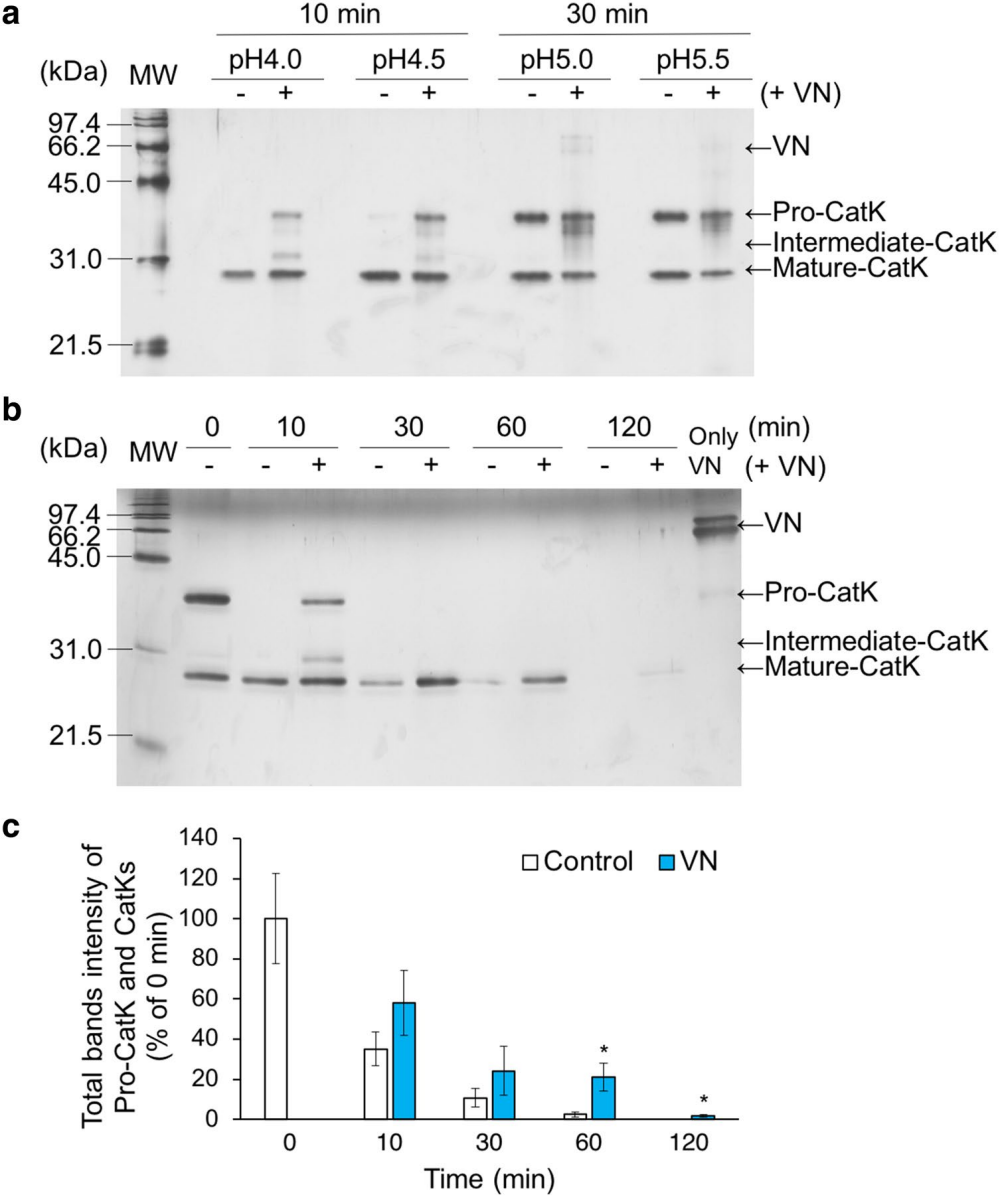
Effects of VN on autoprocessing of pro-CatK. (**a**) Autoprocessing of pro-CatK to mature-type CatK in pH 4.0–5.5, with or without 2 μM hVN, for indicated times. (**b**) Time course of pro-CatK activation at pH 4.0, with or without 2 μM hVN. (**c**) Total band intensities, including pro-CatK, intermediate-CatK, and mature-CatK were quantified using Image J. *p < 0.05 compared with control via an unpaired *t* test. n = 3. Values represent the mean, and error bars represent the SE. Experiments were performed independently at three times.

CatK is activated from a precursor, pro-CatK, via an intermediate under acidic conditions by N-terminal autoprocessing. We evaluated the effects of VNs on the autoprocessing of pro-CatK by reducing the molecular weight of pro-CatK (35.3 kDa) to CatK (~ 26 kDa) using SDS-PAGE. In the absence of VN, pro-CatK was completely transformed to mature-CatK after incubation for 10 min at pH 4.0, while a small amount of pro-CatK remained after incubation for 10 min at pH 4.5 (Fig. 5a). In the presence of VN, pro-CatK and intermediate-CatK were detected after incubation for 10 min at pH 4.0 and pH 4.5. About half of pro-CatK was activated to the mature type and the other half remained as pro-CatK, and there was no significant difference between incubation for 30 min at pH 5.0 and 5.5, with and without hVN. Thus, hVN inhibited the autoprocessing of pro-CatK at pH 4.0 and 4.5. The time course of hVN-mediated effects on pro-CatK activation at pH 4.0 was examined (Fig. 5b). At 10 min after incubation, pro-CatK was completely activated to mature-CatK in the absence of VN, but some of pro-CatK remained inactivated when hVN was present. At 30 and 60 min, the activated mature-CatK content was higher in the presence of VN than in the absence of VN. VN increased the levels of pro-CatK, intermediate-CatK, and mature-CatK (Fig. 5c). These results suggest that VN not only inhibits pro-CatK activation but also inhibits degradation of the mature type.

### Binding of VN to collagen and CatK

**Figure 6:**
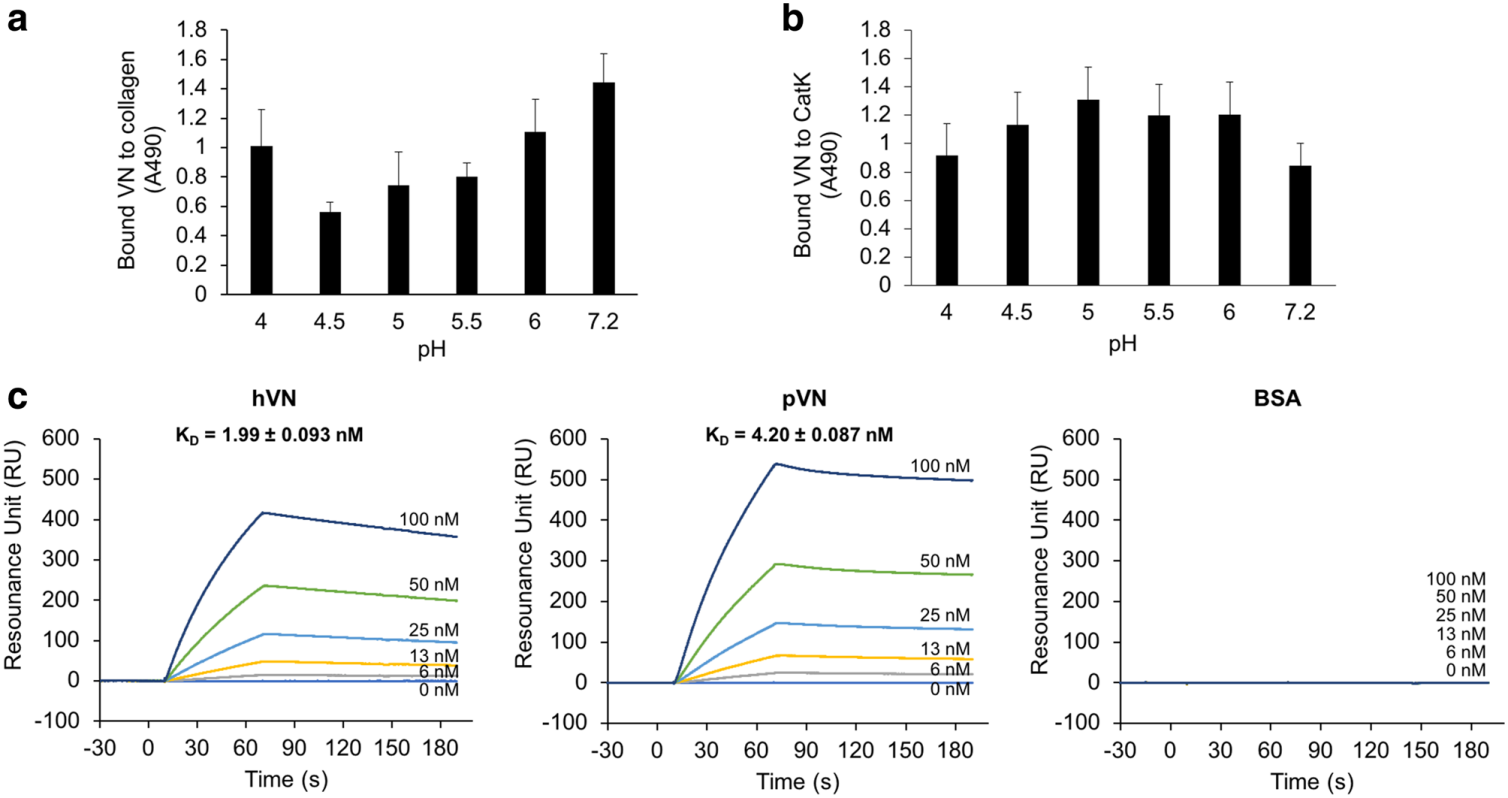
Binding of VNs to collagen and CatK. (**a**) The binding of hVN to porcine type I collagen at pH 4.0–7.2 by ELISA. (**b**) The binding of hVN to CatK at pH 4.0–7.2 by ELISA. (**c**) The SPR sensorgrams hVN binding, pVN, and BSA to immobilized CatK on the CM5 chip. hVN, pVN, and BSA concentrations injected during SPR experiments were 0, 6, 13, 25, 50, and 100 nM. Each K_D_ was calculated by fitting to a 1:1 kinetics binding model. Values represent the mean, and error bars represent the SE. n = 3. Experiments were performed independently three times.

VN’s binding to collagen and CatK at pH 4.0–7.2 was analyzed by ELISA. The binding of VN to collagen showed a U-shaped curve, with a maximum binding pH of 7.2 and low binding pH of 4.5–5.5 (Fig. 6a). Conversely, VN’s binding to collagen showed an inverted U-shaped curve, with a pH of 5.0 for maximum binding and a pH of 7.2 for low binding (Fig. 6b).

Additionally, VN specifically binds to collagen^5^, but the interaction between VN and CatK has not been reported. To determine whether VNs can specifically bind to CatK, real-time binding analysis by Surface plasmon resonance (SPR) was performed using immobilized CatK on the chip and different dilutions of hVN, pVN, and bovine serum albumin (BSA) (Fig. 6c). Both VNs showed affinities to CatK but not to BSA, suggesting that VN’s binding to CatK is specific. The dissociation constants (K_D_) of hVN and pVN to CatK were 1.99 ± 0.093 nM and 4.20 ± 0.087 nM, respectively. These results suggest that VN preferentially binds to collagen under neutral conditions and that VN binding targets under acidic conditions switch from collagen to CatK.

### Effects of VN glycosylation

**Figure 7:**
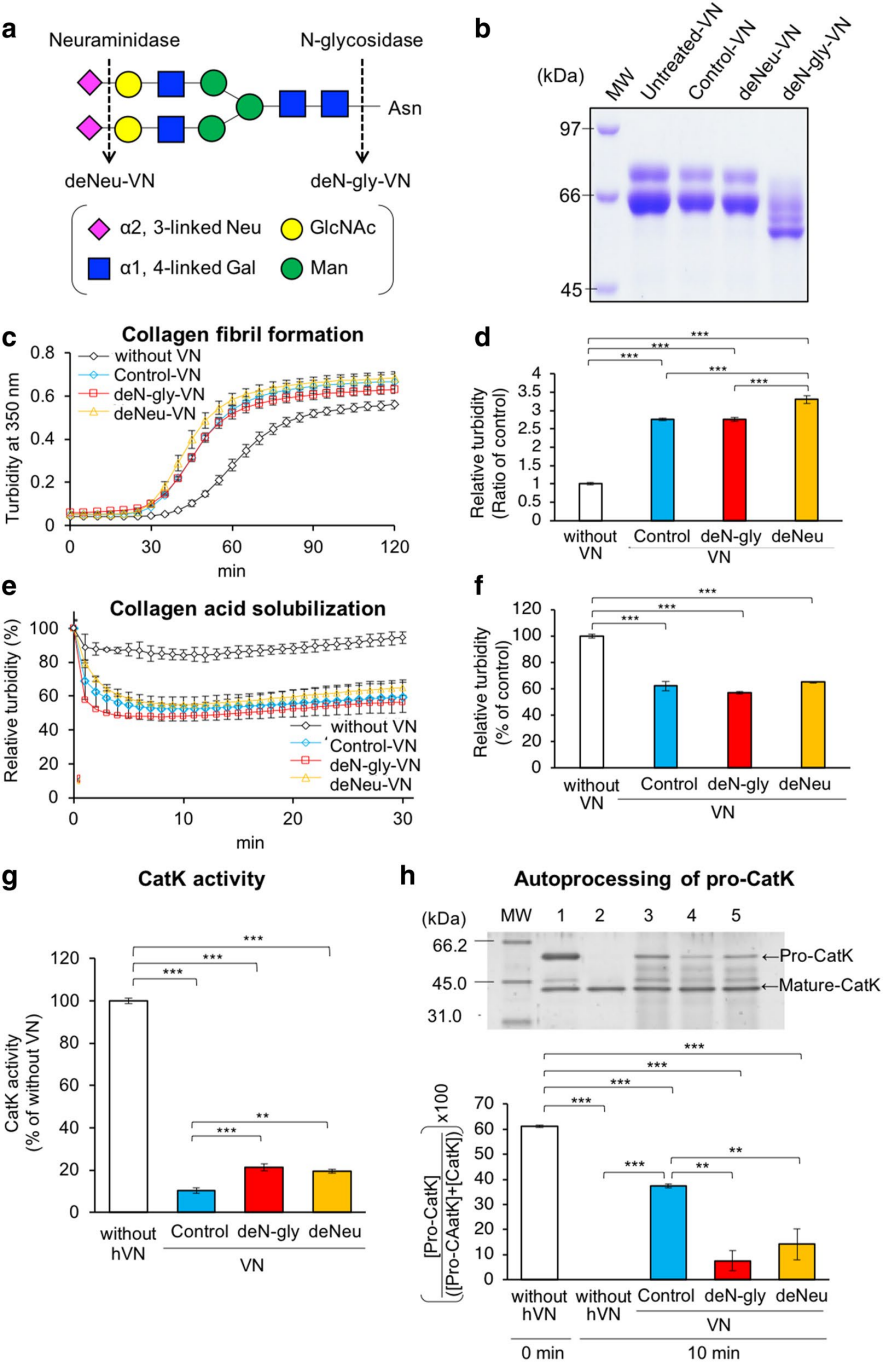
Effects of glycosylation of VNs on collagen fibril formation, acid solubilization, CatK activity, and autoprocessing of pro-CatK. (**a**) Cleavage sites of enzymatic deglycosylation in the structure of the major N-glycan on hVN. (**b**) Glycosidase-treated hVNs (4 μg per lane) were loaded in each lane of 7.0% polyacrylamide gel and subjected to SDS/PAGE under reducing conditions with 2-mercaptoethanol. The gel was stained with Coomassie Brilliant Blue (CBB). (**c**) Collagen fibril formation; collagen-formed fibrils during incubation for 2 h at 37 °C and pH 7.2, with or without 0.1 mg/mL hVNs, and measured at 350 nm, as described in Materials and Methods. (**d**) Relative turbidity (ratio of control) at 50 min in (**c**). n = 4. (**e**) Collagen acid dissolution: collagen-formed fibrils during incubation for 2 h at 37 °C at pH 7.2, with or without 0.1 mg/mL hVNs, then 1 M pH 5.0 buffer was added, and the absorbance of the sample at 350 nm was measured as described in “Materials and methods”. (**f**) Relative turbidity (% of control) at 10 min in (**e**). n = 4. (**g**) CatK activity measured at 37 °C for 60 min at pH 5.5, with or without 1 μM hVNs. CatK activities are shown as % of those without hVN. n = 4. (**h**) Pro-CatK activation analysis using SDS-PAGE. Pro-CatK was incubated with or without 2 μM hVNs at 37 °C for 10 min at pH 4.0, as described in Material and Methods. Lane 1, pro-CatK before incubation in pH 4.0; Lane 2, pro-CatK after incubation without VN for 37 °C for 30 min in pH 4.0; Lane 3, pro-CatK after incubation with 2 μM control-VN for 37 °C for 30 min in pH 4.0; Lane 4, pro-CatK after incubation with 2 μM deN-gly-VN for 37 °C for 30 min in pH 4.0; Lane 5, pro-CatK after incubation with 2 μM deNeu-VN for 37 °C for 30 min in pH 4.0. Quantifying bands of SDS-PAGE using ImageJ. Pro-CatK activation is shown as ([Pro-CatK]/([Pro-CatK] + [CatK])) × 100. [ ] is the intensity of a band on SDS-PAGE. n = 3. *p < 0.05; **p < 0.01; ***p < 0.001 compared with control-VN by one-way ANOVA with Tukey's post-hoc test. Experiments were performed independently 3–4 times.

hVN has three N-glycosylation sites, and the structures of their carbohydrate chains mainly comprise two or three bi-antennary sialylated complex-type N-glycans, as shown in Fig. 7a^36^. pVN has similar structures of N-glycans^37^. We prepared deN-gly-VN and deNeu-VN by incubation with N-glycosidase and neuraminidase, respectively. Deglycosylation of VN was confirmed by reduced molecular weight, as described previously (Fig. 7b)^5,9–11^. We investigated the effects of hVN glycosylation on collagen fibril formation, collagen acid solubilization, CatK activity, and autoprocessing of pro-CatK.

The increase in turbidity due to collagen fibril formation was accelerated by all VNs, and deNeu-VN caused slightly higher increase than the control- and deN-gly-VNs (Fig. 7c,d). On the other hand, acid-induced reduction in relative turbidity was enhanced for all VNs, including the control, deN-gly-VN, and deNeu-VN, although differences between the three VNs were not significant (Fig. 7e,f). Control-, deN-gly-, and deNeu-VN inhibited CatK activity by 10.4%, 21.3%, and 19.5%, respectively, compared to that without VN (Fig. 7g). This inhibition of CatK activity by control-VN was stronger than that by deN-gly- and deNeu-VNs. Figure 7h shows the result of autoprocessing of pro-CatK with SDS-PAGE, and the remaining pro-CatK content was evaluated using ImageJ. In the absence of hVN, the remaining pro-CatK content was reduced from 61.2 to 0% after incubation for 10 min. In contrast, the remaining pro-CatK contents were 37.4%, 7.5%, and 14.2% after 10 min incubation with control-, deN-gly-, and deNeu-VNs, respectively. These results indicate that control-VN inhibited pro-CatK autoprocessing more effectively than deN-gly-VN and deNeu-VN.

**Figure 8:**
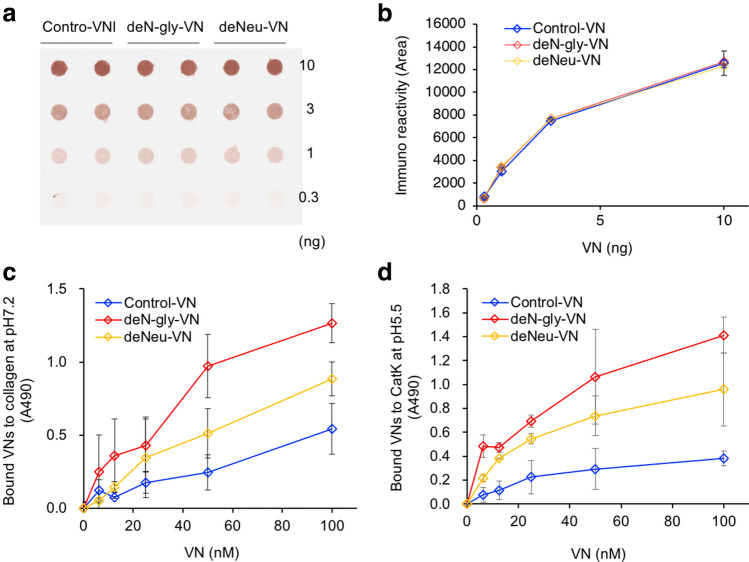
Effects of glycosylation of VNs on binding to CatK. (**a**) Immunoreactivity of glycosidase-treated hVNs by dot-blotting. hVNs (0.3, 1, 3, 10 ng) were dot-blotted onto PVDF and immunostained with rabbit anti-VN antibody and HRP-anti-rabbit secondary antibody as described in Methods. (**b**) Densitometric analysis of spots on blotting membrane by ImageJ. (**c**) The binding of glycosidase-treated hVNs to porcine type I collagen at pH 7.2 by ELISA. (**d**) The binding of glycosidase-treated hVNs to CatK at pH 5.5 by ELISA. Data shown represent the mean ± SD. Experiments were performed independently three times.

VNs’ binding to collagen or CatK was assayed by ELISA. Since glycosylation of VNs may affect immunoreactivity, the reactivity of glycosidase-treated VNs was examined by dot-blotting before binding assay. It was confirmed that all VNs had almost the same immunoreactivity (Fig. 8a,b). By ELISA binding assay, VNs bound to collagen in the order of deN-gly-VN > deNeu-VN > Control-VN (Fig. 8c). In effects of VN glycosylation, VNs’ binding to CatK was similar to VNs’ collagen binding (Fig. 8d).

### VN degradation by CatK

**Figure 9:**
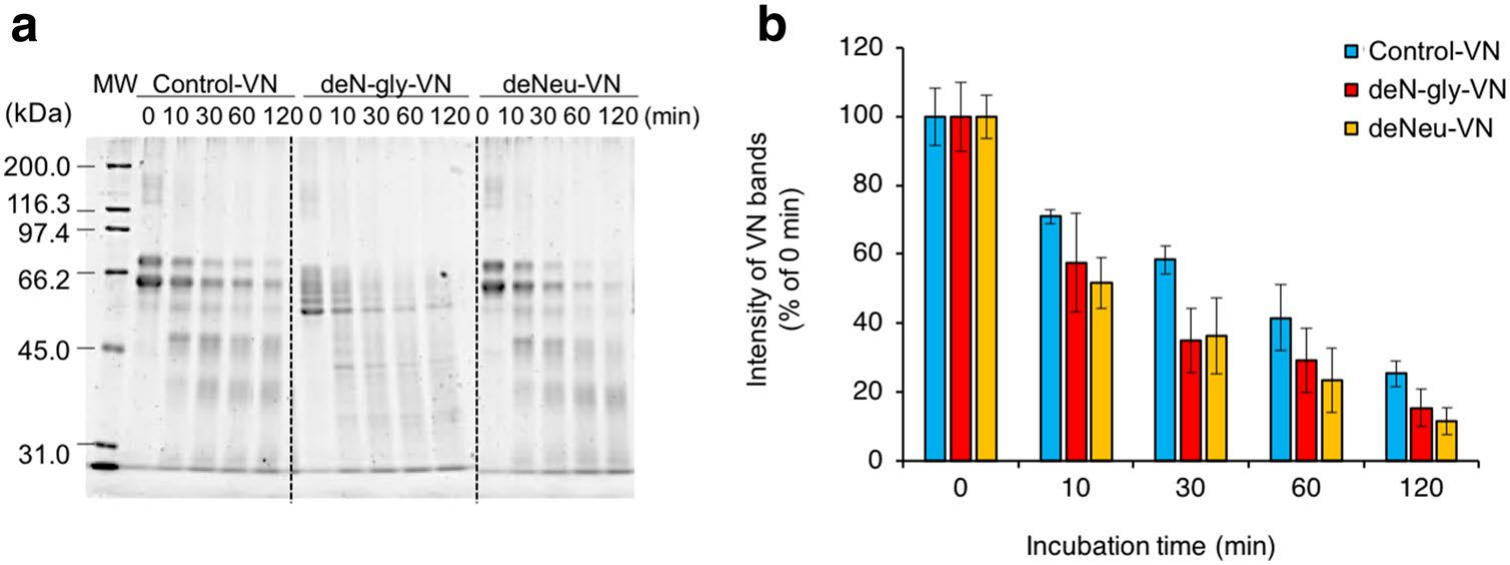
Degradation of VNs by CatK. (**a**) CatK (10 nM) and hVNs (2 μM) were incubated at 37 °C for indicated times and E-64 was added to stop the reaction by CatK. The degradation of hVN in samples was analyzed by SDS-PAGE as described in Methods. (**b**) Quantifying bands of SDS-PAGE in (**b**) Quantifying bands of SDS-PAGE in (**a**) using ImageJ. Degradation of hVNs by CatK are shown as intensity of hVN bands (% of 0 min). n = 3. deN-gly-VN and deNeu-VN compared with control-VN via one-way ANOVA with Tukey's post-hoc test. Experiments were performed independently three times.

SDS-PAGE for the autoprocessing products of pro-CatK (Fig. 5a,b) revealed that VNs disappeared after incubation with pro-CatK, indicating that they can be degraded by CatK. In this study, VN degradation by CatK was demonstrated by reduced VN molecular weight, as determined using SDS-PAGE (Fig. 9a). The molecular weights of control hVN and its degraded form were 75 and 65 kDa, respectively, and those of deN-gly and deNeu-VNs were reduced due to cleavage of N-glycosidase F and neuraminidase, respectively (Fig. 7b). The control-, deN-gly-, and deNeu-VNs were time-dependently degraded by 10 nM CatK. There was no significant difference in the contents of undegraded VN among the three VNs due to incubation with 10 nM CatK (Fig. 9b). VN was not degraded after 120 min of treatment with 1 nM CatK but was completely decomposed within 10 min of treatment with 100 nM CatK. VNs were degraded by 10, 30, and 100 nM CatK, and no significant difference was found between the control, deN-gly, and deNeu-VNs (Supplementary Figure S2). These results suggest that N-glycosylation of VNs is dispensable, but enhances their inhibitory effects on CatK activity and pro-CatK activation, and that sialylation of VN is also not essential but enhances collagen fiber formation.

## Discussion

**Figure 10:**
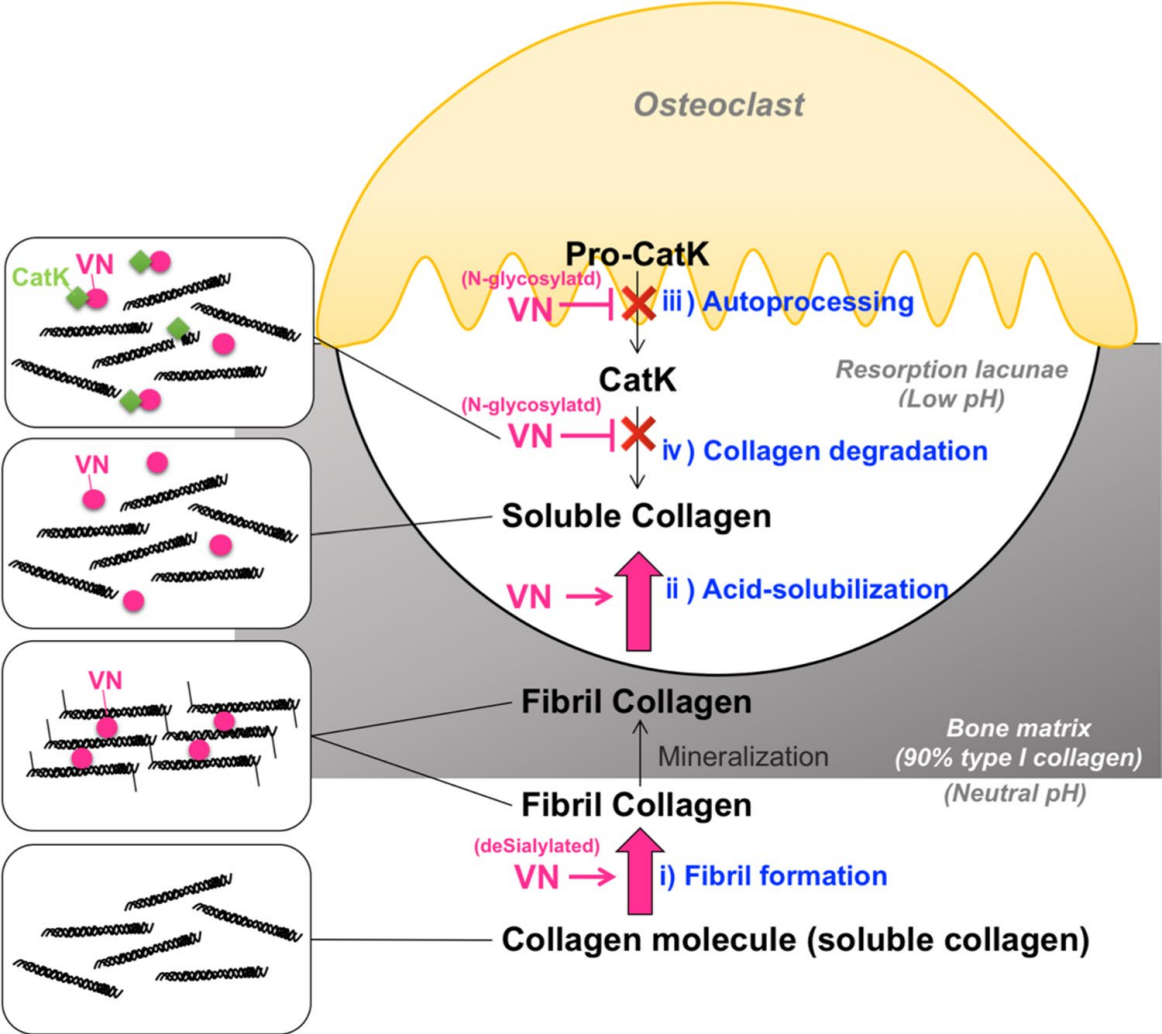
Schematic illustration of the effects of VN on collagen fibril formation and collagen degradation by CatK. Under neutral conditions, VN promotes i) collagen fibril formation. Under acidic conditions (such as acidification by osteoclasts), ii) VN forms collagen fibers with high acid solubility. VN inhibits iii) autoprocessing of pro-CatK to CatK, and iv) CatK activity. Desialylation of VN enhances i) promotion of collagen fibril formation, and N-glycosylation of VN enhances iii) inhibition of autoprocessing of Pro-CatK and iv) inhibition of CatK activity.

This is the first report of VNs interacting with CatK, which also demonstrates CatK inhibition by these biomolecules. The results of this study and the inferences from the results are summarized in a schematic (Fig. 10). i) VN accelerates collagen fibril formation under neutral pH conditions (Fig. 1). ii) Under acidic conditions, collagen fibers formed with VNs were readily acid soluble (Fig. 3) and (iii–iv) VNs inhibited autoprocessing of pro-CatK (Fig. 5a) as well as CatK activity at concentrations 240–640 times lower than blood levels (Fig. 4). VN, bound to collagen at neutral pH, switches binding to CatK to inhibit its activity around pH 5.0 (Fig. 6). Although VN glycosylations are not essential for the regulation of these activities, VN desialylation is effective for collagen fibril formation, and its N-glycosylation is effective for inhibition of both pro-CatK autoprocessing and CatK activity (Fig. 7).

The promotion of collagen fibril formation by VNs was expected, because of the adhesive properties of VNs. However, it was surprising that the collagen fibers formed with VNs were easily acid-dissolved (Fig. 3a,b). Most VNs were in the fraction of solubilized collagen (Fig. 3c). VN’s collagen-binding activity is high at neutral pH and low around pH 5.5, which is CatK optimum pH (Fig. 6a). In contrast, the VN’s CatK binding activity is high around pH 5.0 and low at neutral (Fig. 6b). These results suggest that VN crosslinks collagen fibers by strongly binding collagen and promoting the formation of collagen fibers at neutral pH. And that the change to acidic pH weakened the crosslinking of collagen fibers by VN and promoted solubilization of collagen fibers. Then, VN switches binding to CatK to inhibit its activity (Fig. 10, In the left four frames).

As shown in Fig. 4e,f, Dixon plots demonstrate that the CatK inhibition by both hVN and pVN is competitive. Figure 9 show that VN was degraded by the high concentration of CatK, suggesting that VN is a substrate for CatK; this is consistent with the results of Fig. 4e,f that CatK inhibition by VN is competitive inhibition.

**Figure 11:**
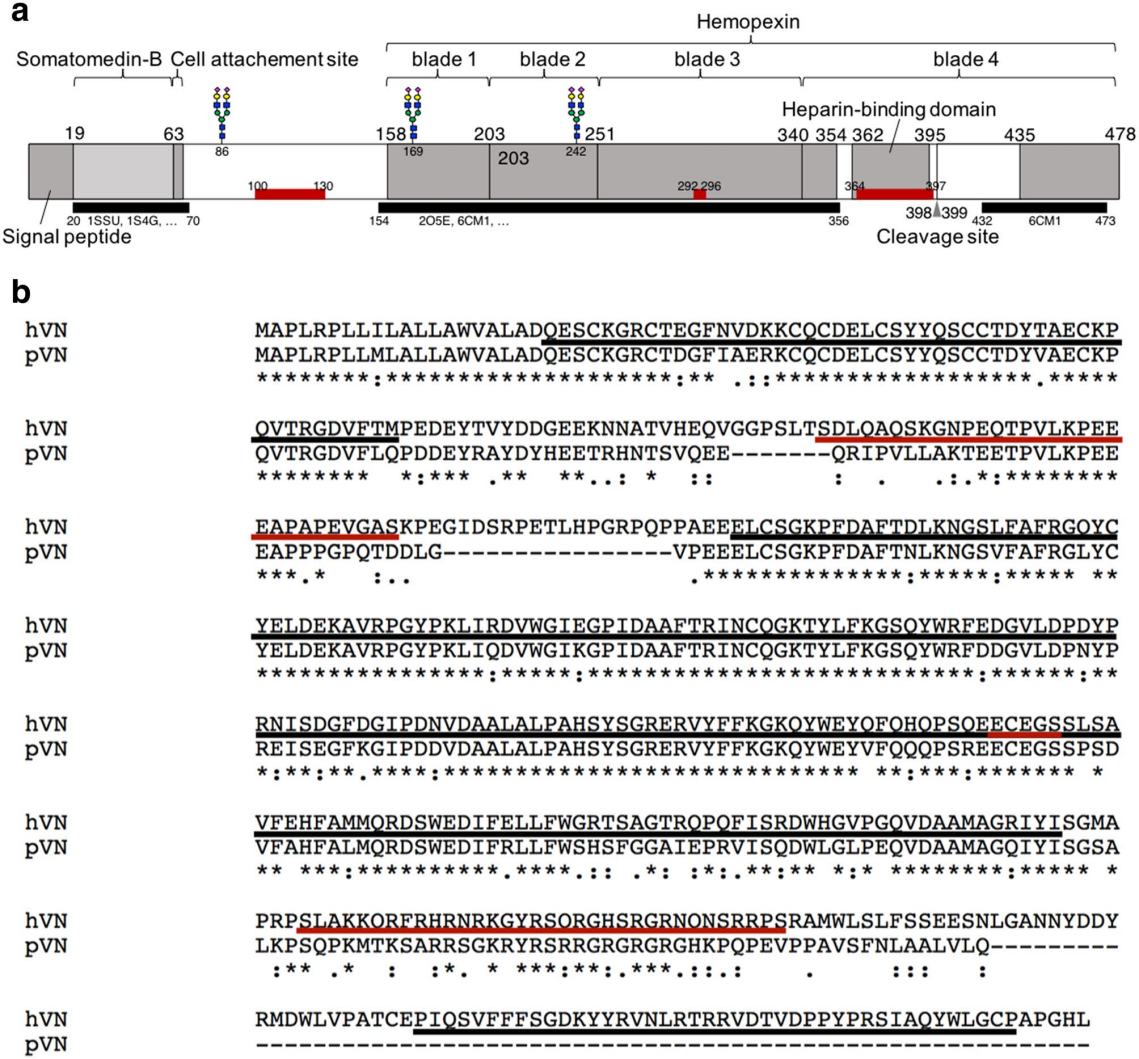
A diagram of hVN and its intrinsically disordered region prediction. (**a**) Red bars are the predicted intrinsically disordered regions, gray regions were depicted based on Uniprot^66^ entry P04004, and black bars are the regions with known three-dimensional structure in Protein DataBank^67^. N-glycosoylation sites at Asn-86, 169 and 242 are shown by a schematic carbohydrate. The prediction method is described in the Discussion. (**b**) Multiple alignment was performed by ClustalW^68^ (ver. 2.1) using the NCBI protein sequence of EAW51082.1 for hVN and BAA09616.1 for pVN. A black bars are the regions with known three-dimensional structure in the Protein Databank. A red bars are the predicted intrinsically disordered regions.

VN has several functional domains: the somatomedin-B, cell attachement site (the R-G-D motif), a connecting region, and hemopexin consist of four blades^38^ (Fig. 11a). Hemopexin blade four is interrupted by heparin-binding domain, responsible for VN’s binding to several other ligands, including collagen^39^, sulfated glycosaminoglycans (GAGs)^40,41^, and integrin α_V_β_5_^42^ (not integrin αvβ3). hVN and pVN have 67–71% amino acid sequence identity with respect to the common domain organization and ligand-binding activities^43^. pVN has an unusually low molecular weight among mammalian VNs including hVN. This is ascribed to a lack of 22 amino acids in the connecting region and a lack of an 80 amino acid fragment from the C-terminal end compared to hVN, but pVN has all four functional domains^43^. In this report, we show that pVN and hVN are comparable in CatK inhibition. It is suggested that the CatK binding site of VN is sufficient for the sequence contained in pVN.

hVN’s three-dimensional structure has been determined partially, its full length has not been solved, suggesting the existence of an intrinsically disordered region. It is generally known that the intrinsically disordered region interacts with other molecules undergoing a disorder-to-order transition upon binding^44^. hVN’s hemopexin domain has disordered regions where collagen binds^39^ and *Yersinia pestis* outer membrane protein Ail interacts^38^. To find the CatK interaction sites in hVN, we predicted intrinsically disordered regions using the following four methods’ consensus: PONDR^45^ (ver. 2002, 2003, http://www.pondr.com/), DisEMBL^46^ (ver. 1.5, http://dis.embl.de/), PrDOS^47^ (ver. 2006, http://prdos.hgc.jp/cgi-bin/top.cgi), and modified FoldIndex^48^, of which the modified version was initially developed by Yura and Hayward^44^. We used a website built by the developer for PONDR, DisEMBL, and PrDOS. For DisEMBL, a region predicted as Hot-loop was employed in this analysis. For FoldIndex, the in-house program was used. The disorder values were calculated along the amino acid sequence in the sliding window with size 19, and a residue with a value less than zero was defined as disordered residue. Furthermore, multiple sequence alignment was performed by ClustalW (ver. 2.1, https://www.genome.jp/tools-bin/clustalw) using the protein sequence of hVN and pVN; both VNs inhibit CatK activity. As shown in Fig. 11a,b, red bars (aa 100–130, 292–296, 364–397) in hVN are the predicted intrinsically disordered regions, and the three regions were conserved in pVN. From these computational analyses, we conjectured that CatK binds to the disordered region in the hemopexin domain.

CatK, an attractive therapeutic target for diseases with excessive bone resorption such as osteoporosis and osteoarthritis^26–32^, is a major lysosomal protease that accounts for 98% of total cysteine protease activity in osteoclasts and is responsible for the degradation of bone matrix proteins^49^. CatK inhibition circumvents the bone coupling mechanism and has excellent effects on osteoporosis, thus suppressing bone resorption without reducing bone formation^50,51^.

Findings of this study regarding CatK activity could be relevant to treating human osteoporosis, particularly as we demonstrated the importance of the VN–CatK interaction between purified molecules in vitro*.* Notably, because chemical compounds such as odanacatib^27^, ONO-5334^28,29^, and MIV-711^31,32^, which show CatK inhibitory effects in the same in vitro assay used in this study, effectively alleviated osteoporosis or osteoarthritis in clinical trials. Other characteristics of VN, which include promoting collagen fibril formation as found in this study—and enhancement of osteoblast adhesion^52^, might be beneficial in bone formation. Unfortunately, odanacatib development has been discontinued, due to the reports of increased stroke risk, although it has been shown to be effective in clinical trials^26,30^. MIV-711 has beneficial effects on bone and cartilage with a reassuring safety profile in humans in clinical trials^31,32^. The present study identified VN as an alternative biomolecule with CatK inhibitory activity. VN may be a key molecule in the treatment of osteoporosis and osteoarthritis, although further research is required in future.

The effects of VN on CatK activity and autoprocessing using osteoclasts, which predominantly express CatK, are particularly interesting. For both cell- and animal-based assays, it is necessary to identify the active site of VN showing CatK inhibitory activity. VN is an adhesive glycoprotein and enhances cell adhesion as well as spreading of almost all cells, including osteoclasts and osteoblasts, in cell-based assays^12,53^. The adhesive properties of VN are likely attributable to the RGD-motif that binds integrin, especially osteoclasts highly expressing the VN receptor α_v_β_3_ integrin^12^. In the study using osteoclasts and osteoblasts, it is presumed that the true effect of CatK cannot be evaluated, because the RGD-motif in VN largely contributes to the observed effects. Therefore, we aimed to determine the active sites of VN with CatK inhibitory and collagen fibril formation promoting activities. In the near future, we plan to investigate how (sugar) fragments or (sugar) peptides affect osteoclasts and osteoblasts, and an osteoporosis- or osteoarthritis-based mouse model to determine whether the CatK inhibitory effects of VN can be applied in treating osteoporosis and/or osteoarthritis.

Recently, it was reported that VN-derived peptides that do not contain RGD-motif reversed ovariectomy-induced bone loss via the regulation of osteoblast and osteoclast differentiation^14^. The VN-derived peptide was identified by screening for agents promoting the attachment and spread of osteogenic cells but restraining osteoclast differentiation and function in vivo. We suspect that the restraining effects of the VN-derived peptide on osteoclasts may be mediated by CatK, although there is no mention of CatK in the concerned report.

In this study, the measurement of collagen fibril formation based on turbidity showed that VNs shortened the T lag and increased turbidity for 60 min (Fig. 1c), suggesting that they promote the collagen fibrosis rate. In addition, VNs increased the plateau value (Fig. 1c). A previous report has shown that turbidity mostly increases during collagen self-assembly due to increasing fibril thickness^33^ and increased molecular weight of the aggregates^34^. Analysis of collagen morphology using SEM showed that VN brought the collagen fibers into close contact with each other to increase the molecular weight and the thickness, consistent with previously reported interpretations.

Bovine and skin collagen from both porcine and bovine are widely utilized for industrial purposes. Since porcine collagen resembles human collagen, it does not cause a significant allergic response in humans, whereas ~ 3% of the population is allergic to bovine collagen^54–56^. Therefore, it is likely that pVN accelerates collagen fibril formation in humans.

Human, rabbit, and mouse VNs have completely conserved potential N-glycosylation sites, as deduced from protein sequences^57–59^. pVN and hVN have two and three potential N-glycosylation sites, respectively. Two potential glycosylation sites in pVN and hVN correspond to the same positions; however, pVN lacks one site on the C-terminal side that correspond to the third glycosylation site present in hVN^60^. The major oligosaccharides in pVN and hVN are common structures belonging to the biantennary complex N-glycan containing 1–3 mol sialic acid, and pVN has more core fucosylation than hVN^36,37^. In this study, hVN glycosylation is primarily important for enhancing the effects on CatK activity and autoprocessing of pro-CatK, whereas there is little effect of hVN glycosylation on VN degradation by CatK and acid solubilization (Figs. 7, 9). These results suggested that the site and/or mode of hVN that inhibits CatK activity and its activation is different from the ones collagen-binding of hVN.

VN’s binding to collagen and CatK was both deN-gly-VN > deNeu-VN > Control-VN in order of strength (Fig. 8). In our previous study, VN is multimerized, and its molecular weight is larger in the order of deN-gly-VN > deNeu-VN > Control-VN^10^. It is considered that de-glycosylated VN enhances CatK binding activity due to its multivalent effect. Then, VN’s larger multimerization by deglycosylation may prevent VN from binding to the substrate binding site of CatK. In the interaction between VN and collagen, the slight enhancement of collagen fibril formation by deNeu-VN (Fig. 7d) may be due to the shift of VN pI to alkali after neuraminidase treatment^9^. Because the molecular organization within collagen fibrils is determined by numerous non-covalent interactions between collagen molecules, particularly charge-charge interactions^61^.

Previous studies have shown that complex N-glycan is important for bone formation and bone diseases^62^. The congenital disorder of glycosylation type IIa (CDG-IIa) has a deficiency in complex N-glycans, which is commonly observed in several diseases including osteopenia and kyphoscoliosis^62,63^. Mice lacking the *Mgat2* gene were deficient in GlcNAcT-II glycosyltransferase activity and complex N-glycans, which resulted in severe gastrointestinal, hematologic, and osteogenic abnormalities^43^. Previously, there have been no published reports regarding the effects of VN glycosylations on bone diseases. In this study, we should show for the first time the importance of VN glycosylation in relation to bone diseases.

This study also revealed that VN is degraded by CatK (Fig. 9). Glycosylation of proteins is generally protease resistant; however, deN-gly-VN and deNeu-VN were found to be more likely to be degraded by CatK than control-VN, although the difference was not statistically significant. Furthermore, we confirmed that VN was not degraded by CatK (0.5 nM) in the CatK activity assessment experiment.

In summary, we showed that VN, a glycoprotein in the extracellular matrix and plasma, enhanced collagen fibril formation and inhibited both CatK activity and autoprocessing of pro-CatK in vitro. Enzymatic deglycosylation of VN attenuated the inhibitory effects of CatK. These data suggest that glycosylated VN may prevent bone destruction through CatK inhibition and that VN could serve as a therapeutic agent for osteoporosis or osteoarthritis. Furthermore, these findings may provide clues to elucidate the mechanism of bone formation and dissolution, and may help to understand the side effects caused by currently used CatK inhibitors^30^.

## Methods

### Materials

Human plasma (each, citric acid) and porcine plasma were purchased from Cosmo Bio Co., Ltd. (Tokyo, Japan) and Tokyo Shibaura Zouki Co., Ltd. (Tokyo, Japan), respectively. N-glycosidase F and neuraminidase were purchased from Roche Molecular Systems, Inc. (Pleasanton, CA, USA). Human recombinant procathepsin K (BML-SE367) and Omni Cathepsin fluorogenic substrate (Z-Phe-Arg-AMC (7-amino-3-methylcoumarin), BML-P139) were purchased from Enzo Life Sciences, Inc. (Farmingdale, NY, USA). Type I collagen from bovine and porcine skin (PSC-1-100, PSC-1-200) were purchased from Nippi, Inc. (Tokyo, Japan). Other sugars and chemical reagents were purchased from Wako Pure Chemicals Inc. (Osaka, Japan) or Nacalai Tesque (Kyoto, Japan).

### Purification and glycosidase treatment of VN

VN was purified from human and porcine plasma by two-step heparin affinity chromatography before and after urea treatment^5,9–11^. A heparin-Sepharose 6B column was prepared using reducing amination methods as described previously^64^. VN (500 μg) was treated with N-glycosidase F (3.3 units) or neuraminidase (16.7 units) at 37 °C for 48 h in 20 mM citrate–phosphate buffer (pH 6.0) containing 1 mM CaCl_2_ and 0.5 mM PMSF^5,9,10^. After glycosidase-treatments, the VNs were dialyzed against 20 mM Tris–HCl-buffered saline (TBS, pH7.2).

### Collagen fibril formation assay

Collagen (2 mg/mL, 50 μL) and VN (indicated concentration, 50 μL) in TBS were combined in a well of an Iwaki 96-well assay plate. After mixing, the collagen solution with or without VN was incubated at 37 °C for 2 h to form fibrils. The turbidity of each well was measured at 350 nm every 5 min using a Cytation3 reader (BioTek)^33,35^. All raw datasets had buffer controls subtracted. The lag phase time (T lag) is defined as the time when the turbidity increased to 0.01, and the plateau value was calculated using Prism 9.0 (GraphPad Software, San Diego, CA, USA).

### SEM

Samples were fixed with 2% paraformaldehyde and 2% glutaraldehyde in 0.1 M cacodylate buffer (pH 7.4) at 4 °C, overnight. The samples were additionally fixed with 1% tannic acid in 0.1 M cacodylate buffer (pH 7.4) at 4 °C for 2 h. After fixation, the samples were washed four times with 0.1 M cacodylate buffer for 30 min each and post-fixed with 2% osmium tetroxide (OsO_4_) in 0.1 M cacodylate buffer at 4 °C for 3 h. The samples were dehydrated once in 50% ethanol for 30 min at 4 °C, once in 70% ethanol for 30 min at 4 °C, once in 90% ethanol for 30 min at room temperature, and then four times in 100% ethanol for 30 min at room temperature. After dehydration, the samples were continuously dehydrated with 100% ethanol at room temperature overnight. The samples were processed once in 50% tert-butyl alcohol/ethanol for 1 h and three times in 100% tert-butyl alcohol for 1 h at room temperature. After dehydration, the samples were frozen at 4 °C and vacuum dried. The samples were coated with a thin layer (30 nm) of osmium using an osmium plasma coater (NL-OPC80A; Nippon Laser & Electronics Laboratory, Nagoya, Japan). The samples were observed via SEM (JSM-7500F; JEOL Ltd., Tokyo, Japan) at an acceleration voltage of 3.0 kV. The pore size area (μm^2^) in SEM images was measured by NIH imaging.

### Acid-solubilization assay of collagen

Collagen (2 mg/mL, 50 μL) and VN (indicated concentration, 50 μL) in TBS were mixed in a well of an Iwaki 96-well assay plate. The collagen solution, with or without VN, was incubated at 37 °C for 2 h to form fibrils. A 1 M pH buffer (1 M acetate buffer pH 4.0–6.0 or Tris–HCl buffer pH 7.2, 10 μL) was added to the collagen fibers. Immediately, the sample in each well was incubated at 37 °C for 30 min, and the turbidity was measured at 350 nm every 1 min using a Cytation3 reader^35^. After turbidity measurements, collagen fibrils in the collagen samples were placed in a new tube with 50 μL of 1 × SDS-PAGE sample buffer. Next, 20 μL of the remaining soluble collagen samples was added to 5 μL of 5 × sample buffer. These samples were heated at 98 °C for 10 min, followed by electrophoresis using 7.0% Tris–glycine gels with 2-mercaptoethanol, and stained with Coomassie Brilliant Blue (CBB).

### CatK activity assay

CatK activity was measured using fluorescence substrates in a 96-well plate^27,28^. Z-Phe-Arg-AMC was dissolved in DMSO at 2 mg/mL and stored at − 80 °C until use. After pro-CatK (5 μM) was autoactivated to CatK in 100 mM acetate buffer at pH 4.0, containing 2.5 mM EDTA/DTT, at 37 °C for 30 min, it was diluted to 4 nM with 5 mM EDTA/DTT. One hundred microliters of the reaction mixture (VN, 10 μM Z-Phe-Arg-AMC, and 0.5 nM CatK in 25 mM pH buffer; acetate buffer, pH 4.0–6.0, or Tris–HCl buffer, pH 7.2, containing 2.5 mM EDTA/DTT) was added to a 96-well assay plate and incubated at 37 °C. Fluorescence emissions were measured at 460 nm after excitation at 380 nm using a Cytation3 reader.

### Pro-CatK autoprocessing assay

Pro-CatK (3 μM) in 100 mM acetate buffer (pH 4.0–5.5), containing 2.5 mM EDTA/DTT, was incubated at 37 °C with or without VN^49,65^. After incubation at 37 °C for 10–120 min, 2 × SDS-PAGE sample buffer was added immediately to the samples, which were then heated at 98 °C for 10 min. The proteins were separated in 15% Tris–glycine gels with 2-mercaptoethanol and stained with EzStain Silver (AE-1360; ATTO Technology, Amherst, NY, USA). The total intensities of both pro-CatK and CatK bands were quantified using ImageJ (NIH, Bethesda, MD, USA).

### ELISA

Collagen and CatK binding activities of VN were assayed by ELISA. For VN collagen-binding, porcine type-I collagen in TBS (10 μg/mL, 100 μL) were immobilized onto wells of a 96 well Immulon 1B plate (Life Technologies Corporation, CA, USA) for 2 h at room temperature. The wells were washed with TBS containing 0.1% tween20 (TBS-T) three times and then blocked with 3% BSA in TBS-T for 2 h at room temperature. hVN (0–100 nM, 50 μL) in 10 mM acetate buffer saline (pH 4.0–6.0) or 10 mM TBS (pH 7.2) added to wells, and followed by incubated overnight at 4 °C. The VN bound to the immobilized collagens were detected with rabbit anti-VN IgG (Cosmo Bio Co. Ltd, Tokyo, Japan, AB605230, LSL-LB-2096-EX, 1:3000) and goat anti-rabbit IgG Fc HRP conjugated (Merck Millipore, AP156P, 1:5000). After washing with TBS-T three times, the HRP was developed using 0.04% *O*‐phenylenediamine in 0.05 M phosphate–citrate buffer (pH 5.0) containing 0.01% H_2_O_2_ (200 μL), stopped by addition of 2 M H_2_SO_4_ (50 μL), and measured at 490 nm using a Cytation3 reader.

For CatK binding of VN, all steps were performed on ice or at 4 °C because CatK was easily degraded at room temperature, and CatK can degrade VN. 96 well Half Area Microplates (Corning, NY, USA) were used, and solution volume was 40% of ELISA for collagen binding of VN. CatK was activated from pro-CatK by the same methods of “CatK activity assay in Methods” and immobilized at 100 nM overnight at 4 °C. After washing with cold TBS-T, the wells were blocked with 3% BSA in cold TBS-T for 3 h at 4 °C. Subsequent operations were performed in the same way as ELISA for collagen binding of VN, except that the room temperature was changed to 4 °C.

### SPR

VN binding to CatK was determined by SPR multicycle analyses by using BiacoreT-100 (GE Healthcare, USA). All experiments were performed at 20 °C. VN (100 nM) was pre-injected on the CM5 chip and the chip was regenerated by 10 mM NaOH to avoid non-specific binding of VN onto the chip. The CatK activated by same methods of “CatK activity assay in Methods” was diluted to 100 nM with 10 mM sodium acetate buffer pH 5.0 and immobilized on the chip by amine coupling method with a flowrate of 10 mL/min following the manufacturer’s instructions. BSA was also immobilized in 10 mM sodium acetate buffer pH 5.5 on another flow cell of the same chip as a reference. Approximately 150 RU of CatK and BSA were immobilized on each flow cell. HBS*–*EP buffer (10 mM HEPES pH 7.4*,* 150 mM NaCl*,* 3 mM EDTA, 0.005% v/v Surfactant P20) was used as the immobilization running buffer.

hVN, pVN, and BSA (0, 625, 12.5, 25, 50, 100 nM) were injected as analytes with a contact time of 60 s, dissociation time of 120 s, and a flowrate of 20 mL/min. The chip was regenerated with 10 mM NaOH for 30 s. Filtrated 10 mM acetate buffer (pH5.5) containing 2.5 mM EDTA/DTT and 0.005% v/v tween 20 was used as the running buffer. The data were determined kinetic constants for the interaction using the 1:1 kinetic binding model in the Biacore T100 evaluation software.

### Dot-blotting

Three-fold dilution series of VNs (each 100 μL) were dot-blotted onto polyvinylidene fluoride membranes (PVDF) and blocked with 3% BSA in TBS-T, then cut into lanes and reacted with the rabbit anti-VN IgG and goat anti-rabbit IgG Fc HRP conjugated at room temperature for 2 h each. The spots were visualized by ECL reagents (GE Healthcare ECLTM Western Blotting Detection Reagents). Chemiluminescence was detected using an ImageQuant LAS4000 mini imager (GE Healthcare). The plotted signal intensities were calculated using ImageJ (NIH) software.

### VN degradation by CatK

CatK was autoactivated from pro-CatK (5 μM) upon incubation in 100 mM acetate buffer (pH 4.0), containing 2.5 mM EDTA/DTT, at 37 °C for 10 min. CatK was diluted with ice-cold 200 mM acetate buffer (pH 5.5), containing 2.5 mM EDTA/DTT, into 2, 20, 60, and 200 nM solutions, and mixed with 4 μM VN in a 1:1 ratio. The mixed solutions were incubated at 37 °C for 0, 10, 30, 60, or 120 min for CatK to degrade VN. After incubation, ice-cold E-64 (inhibitor of proteinases including CatK; final concentration: 1 μM) and 2 × SDS-PAGE sample buffer were added to the solutions. The samples were immediately heated at 98 °C for 10 min. The samples were electrophoresed using 7% Tris–glycine gels with 2-mercaptoethanol and stained with SYPRO Ruby gel stain. The protein bands were detected using ImageQuant LAS 4000 (GE Healthcare, Chicago, IL, USA). ImageJ (NIH) was used to quantify the intensity of the VN bands corresponding to the molecular weight of the undegraded VN at an incubation time of 0 min.

### Statistical analyses

The results are expressed as the mean ± standard error (SE). For statistical analyses of data, unpaired *t* test or one-way analysis of variance (ANOVA) with a post-hoc test was used as appropriate. All analyses were performed using IBM SPSS Statistics version 26.0 (IBM Corp., Armonk, NY, USA).

## Supplementary Information

Supplementary Information 1.
