# Supplementary material for: Regulatory properties of vitronectin and its glycosylation in collagen fibril formation and collagen-degrading enzyme cathepsin K activity
Source: Sci Rep. 2021 Jun 8;11:12023. doi: 10.1038/s41598-021-91353-6 (PMC8187593; doi:10.1038/s41598-021-91353-6)
Supplement: Supplementary file 1 — Supplementary Information 1. [file 41598_2021_91353_MOESM1_ESM.docx]

***Supplementary Material****s*

**Regulatory properties of vitronectin and its glycosylation in collagen fibril formation and collagen degrading enzyme cathepsin K activity**

**Kimie Date^1^*, Hiromi Sakagami^2^**

^1^Institute for Human Life Innovation, Ochanomizu University, 2-1-1 Otsuka, Bunkyo-ku, Tokyo 112-8610, Japan

^2^Faculty of Science, Department of Chemistry, Ochanomizu University, 2-1-1 Otsuka, Bunkyo-ku, Tokyo 112-8610, Japan

E-mail: ^1^date.kimie@ocha.ac.jp, ^2^sakagami.hiromi@ocha.ac.jp

*Corresponding author: Kimie Date (E-mail: date.kimie@ocha.ac.jp)


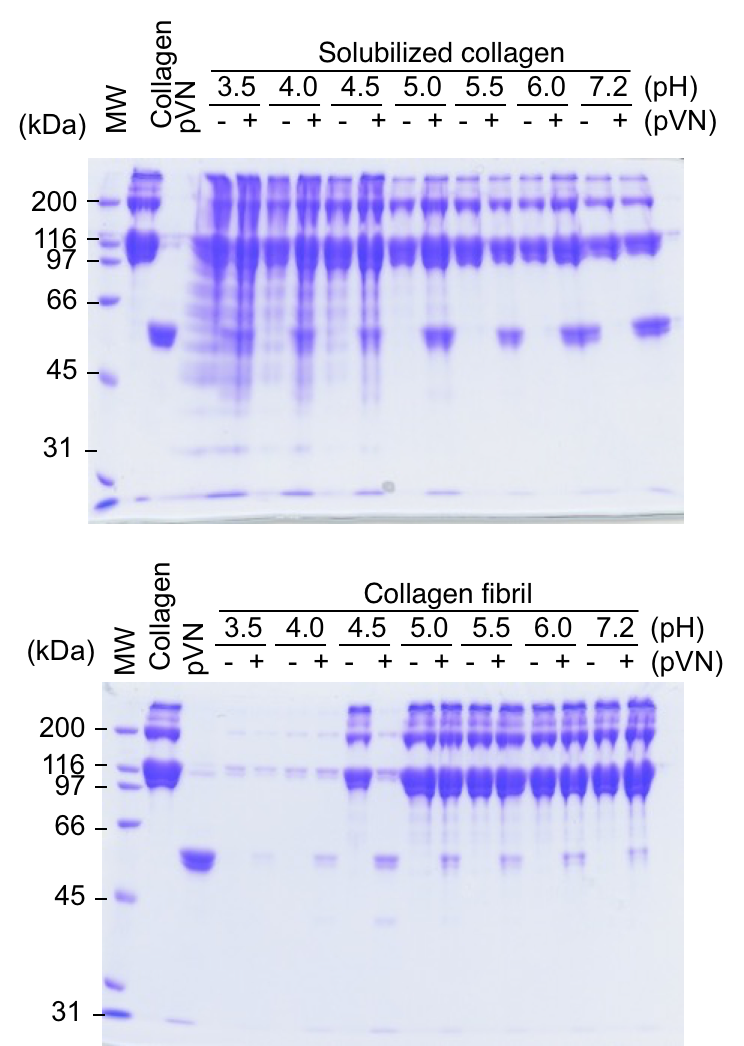


Supplementary Figure S1. Effects of pVN on the acid-solubilisation of collagen fibres. SDS-PAGE of solubilised collagen and collagen fibres from porcine samples after acid-solubilisation at the indicated pH for 30 min. The gel was stained with Coomassie Brilliant Blue. MW, molecular weight; Collagen, 10 μg of untreated collagen; pVN, 2 μg of porcine vitronectin ; −, without 0.1 mg/mL pVN; +, with 0.1 mg/mL pVN.


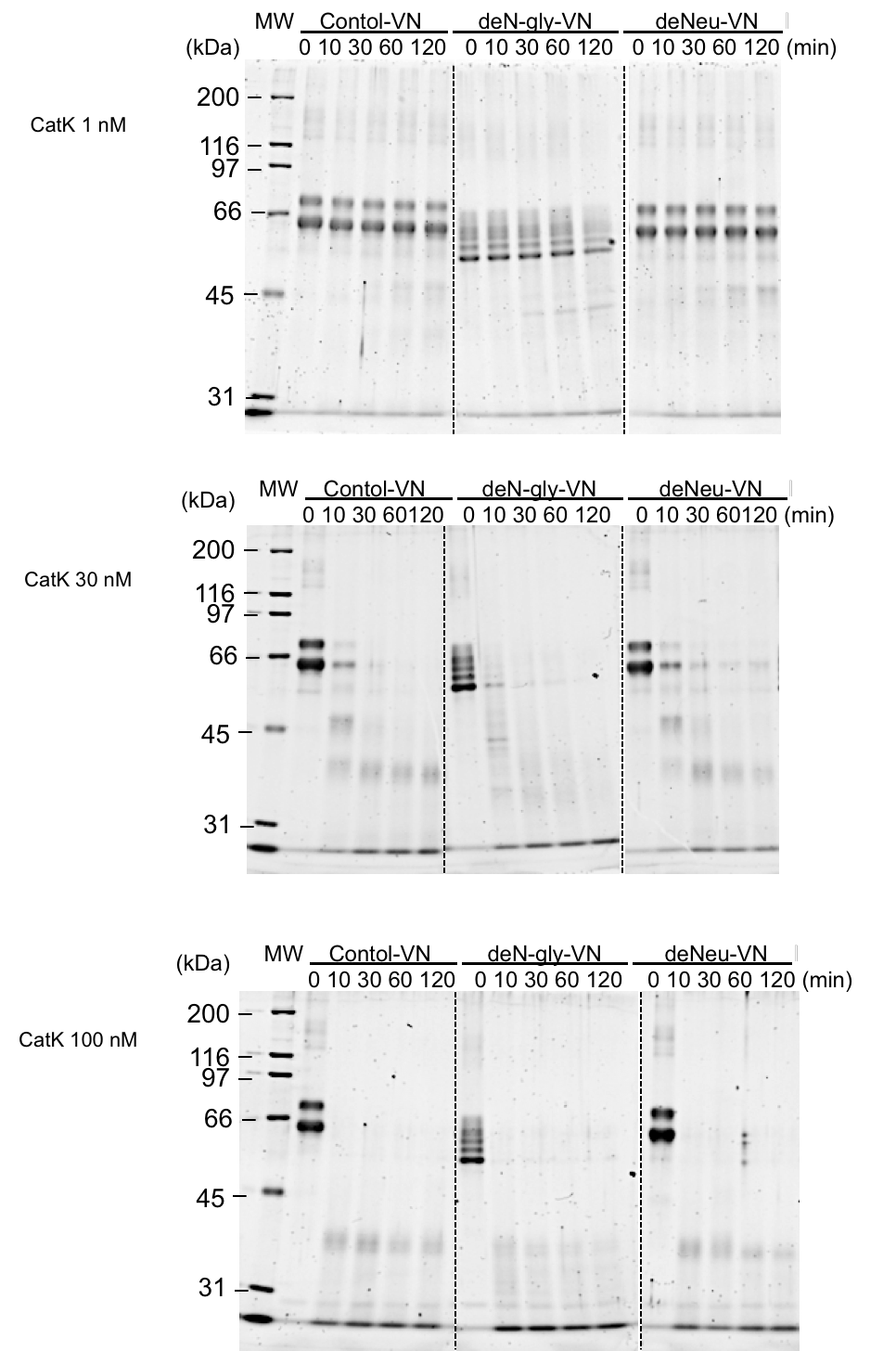


**Supplementary Figure S2. Degradation of VN by CatK.** Non-treated control-VN, and deN-gly-VN and deNeu-VN treated with N-glycosidase and F neuraminidase, respectively, as described in the Methods section, (2 μM each) were incubated with CatK (1, 30, or 100 nM) at 37°C for the indicated times. E-64 was added to stop the CatK-induced reaction. The VN degradation in the samples was analysed via SDS-PAGE. The gel was stained with SYPRO Ruby.
